# Retraction: Demineralized Bone Matrix Combined Bone Marrow Mesenchymal Stem Cells, Bone Morphogenetic Protein-2 and Transforming Growth Factor-β3 Gene Promoted Pig Cartilage Defect Repair

**DOI:** 10.1371/journal.pone.0347913

**Published:** 2026-04-24

**Authors:** The PLOS One Editors

After publication of this article [1,2], concerns were raised regarding results presented in Fig 6. Specifically, in Fig 6E when contrast levels are adjusted, there appear to be multiple repetitive bands and background areas, horizontal and vertical discontinuities, and areas that appear to be discontinuous with their adjacent background regions within the panel.

The authors did not respond to editorial request for comment on these concerns.

In light of the above concerns which call into question the integrity and reliability of the results in [1], the *PLOS One* Editors retract this article.

All authors either did not respond directly or could not be reached.

After publication of this article [1,2], it was also raised that the following panels in [1] appear similar to panels in later Review articles [3,4] by different author groups. These concerns do not contribute to the *PLOS One* Editors’ decision to retract this article.

- Fig 1A in [1] and Fig 1E in [3]
- Fig 9A in [1], Fig 1F in [3], and Fig 11A in [4]
- Fig 9D in [1] and Fig 11B in [4]
- Fig 10 4W, 8W and 12W images in [1] and Fig 4 Pig osteochondral images in [3].
